# CD93 Correlates With Immune Infiltration and Impacts Patient Immunotherapy Efficacy: A Pan-Cancer Analysis

**DOI:** 10.3389/fcell.2022.817965

**Published:** 2022-02-15

**Authors:** Zerui Zhang, Mengli Zheng, Qiang Ding, Mei Liu

**Affiliations:** Department of Gastroenterology, Institute of Liver and Gastrointestinal Diseases, Tongji Hospital of Tongji Medical College, Huazhong University of Science and Technology, Wuhan, China

**Keywords:** CD93, pan-cancer, tumor immunity, TME, immunotherapy

## Abstract

**Background:** The clinical implementation of immune-checkpoint inhibitors (ICIs) targeting CTLA4, PD-1, and PD-L1 has revolutionized the treatment of cancer. However, the majority of patients do not derive clinical benefit. Further development is needed to optimize the approach of ICI therapy. Immunotherapy combined with other forms of treatment is a rising strategy for boosting antitumor responses. CD93 was found to sensitize tumors to immune-checkpoint blocker therapy after the blockade of its pathway. However, its role in immune and ICB therapy across pan-cancer has remained unexplored.

**Methods:** In this study, we provide a comprehensive investigation of CD93 expression in a pan-cancer manner involving 33 cancer types. We evaluated the association of CD93 expression with prognosis, mismatch repair, tumor mutation burden, and microsatellite instability, immune checkpoints, tumor microenvironment, and immune using multiple online datasets, including The Cancer Genome Atlas, Cancer Cell Line Encyclopedia, Genotype Tissue-Expression, cBioPortal, Tumor Immune Estimation Resource database, and Tumor Immune Single-cell Hub.

**Results:** CD93 expression varied strongly among cancer types, and increased CD93 gene expression was associated with poor prognosis as well as higher immune factors in most cancer types. Additionally, the level of CD93 was significantly correlated with MMR, TMB, MSI, immune checkpoints, TME, and immune cell infiltration. Noticeably, our results mediated a strong positive contact between CD93 and CAFs, endothelial cells, myeloid dendritic cells, hematopoietic stem cells, mononuclear/macrophage subsets, and neutrophils while a negative correlation with Th1, MDSC, NK, and T-cell follicular helper in almost all cancers. Function analysis on CD93 revealed a link between itself and promoting cancers, inflammation, and angiogenesis.

**Conclusion:** CD93 can function as a prognostic marker in various malignant tumors and is integral in TME and immune infiltration. Inhibition of the CD93 pathway may be a novel and promising strategy for immunotherapy in human cancer. Further explorations of the mechanisms of CD93 in the immune system may help improve cancer therapy methods.

## Introduction

Noncommunicable diseases, especially cancer, are responsible for most global deaths. Approximately 18.1 million new cancer cases and 9.6 million deaths occurred in 2018 (Bray et al., 2018). With the decline in the effectiveness of traditional cancer treatments, there is an urgent need to develop precise medicine to acquire better clinical outcomes. In recent years, the use of immune-checkpoint inhibitors (ICIs), such as targeting PD-1/PD-L1 or CTLA4, has markedly changed the field of cancer treatments (Passaro et al., 2020). However, there is still a significant portion of patients who showed minimal response to ICIs (Samstein et al., 2019). The tremendous differences between different patients can largely be explained by the complex tumor microenvironment (TME). The TME reportedly has a significant impact on clinical outcomes and response to therapy (Bagaev et al., 2021). It is of great significance for cancer treatments to focus on TME, explore new tumor immune targets, and develop drugs specifically targeting the TME.

CD93 may provide new opportunities for the immunotherapy of the TME. CD93 is a transmembrane protein consisting of an extracellular domain with a C-type lectin domain, five tandem EGF-like repeats, a serine-threonine-rich mucin-like domain, a transmembrane domain, and a short cytoplasmic domain prominently expressed in endothelial cells and some hematopoietic subsets (Langenkamp et al., 2015). The intracellular domain (ICD) of CD93, a regular transcription factor, is deemed to regulate gene expression with other transcription factors (Cokol et al., 2000). As one of the top 20 core genes for angiogenesis in human primary tumors, CD93 has been widely reported to play a critical role in inflammation and angiogenesis (Masiero et al., 2013; Lugano et al., 2018). Gao et al. (2021) demonstrated that a CD93-enriched subset in hematopoietic stem cells and multipotent progenitors with enhanced stem cell properties and CD93 can act as a regulatory factor in their development, revealing its critical role in hematopoiesis. More excitingly, a recent study found that blockade of the CD93 pathway could reduce hypoxia and promote drug delivery on account of vascular normalization, resulting in the enhanced antitumor. Resultingly, the antitumor efficacy of gemcitabine as well as 5-fluorouracil (5-FU) was greatly enhanced. Of particular interest is the fact that blocking of the CD93 pathway improves tumor vascular functions to promote T-cell infiltration and antitumor immunity, thereby sensitizing tumors to immune-checkpoint blocker (ICB) therapy. For instance, both CD4^+^ and CD8^+^ T-cell subsets, as well as the expression of PD-L1, were increased in CD93 mAb–treated tumors, whereas the population of myeloid-derived suppressor cells was reduced (Sun et al., 2021). This study found a safer therapeutic target for tumor vascular normalization and provided a novel remedy for oncotherapy. In addition, there are still many other inspiring studies on CD93. Huang et al. identified CD93 as an interleukin (IL)-17D receptor selectively expressed on group 3 innate lymphoid cells (ILC3s). The ILC3 function was regulated by the IL-17D-CD93 axis at the barrier surfaces, promoting homeostasis and protecting against inflammatory diseases (Huang et al., 2021). Moreover, CD93 was recognized as an important regulator of stemness of leukemia stem cells and a potential therapeutic target and the anti-emetic agent metoclopramide as a drug that blocks CD93 signaling in chronic myeloid leukemia (CML) (Riether et al., 2021). In the proliferating endothelium, the interaction between CD93 and the extracellular matrix regulated cell adhesion, migration, and vascular maturation by activating relevant signaling pathways (Barbera et al., 2021). Overall, all these findings support the central role of CD93 in carcinoma progression and treatment. It is worthwhile to look forward to exploring a brand-new therapeutic target and making greater progress in the research and clinical field via CD93. Thus, comprehensive CD93 analyses based on prognostic, TME, immunological effects, and therapeutic effects across all cancer types are acquired.

Therefore, we employed the most up-to-date data from numerous databases, including TCGA, Cancer Cell Line Encyclopedia (CCLE), Genotype Tissue-Expression (GTEx), cBioPortal, and tumor immune single-cell hub (TISCH), to systematically evaluate the correlation of CD93 with 33 types of cancer. Our results evaluated the connection between CD93 expression and prognosis, tumor mutation burden (TMB), microsatellite instability (MSI), immune checkpoints, TME, immune cell infiltration, and immune-related genes, providing supportive evidence of its vital role in the process of cancer.

## Materials and Methods

### CD93 Expression Analysis

Transcriptome RNA-seq data of CD93 in 33 cancers, which include adrenocortical carcinoma (ACC), bladder urothelial carcinoma (BLCA), breast invasive carcinoma (BRCA), cervical squamous cell carcinoma and endocervical adenocarcinoma (CESC), cholangiocarcinoma (CHOL), colon adenocarcinoma (COAD), lymphoid neoplasm diffuse large B-cell lymphoma (DLBC), esophageal carcinoma (ESCA), glioblastoma multiforme (GBM), head and neck squamous cell carcinoma (HNSC), kidney chromophobe (KICH), kidney renal clear cell carcinoma (KIRC), kidney renal papillary cell carcinoma (KIRP), acute myeloid leukemia (LAML), brain lower grade glioma (LGG), liver hepatocellular carcinoma (LIHC), lung adenocarcinoma (LUAD), lung squamous cell carcinoma (LUSC), mesothelioma (MESO), ovarian serous cystadenocarcinoma (OV), pancreatic adenocarcinoma (PAAD), pheochromocytoma and paraganglioma (PCPG), prostate adenocarcinoma (PRAD), rectum adenocarcinoma (READ), sarcoma (SARC), skin cutaneous melanoma (SKCM), stomach adenocarcinoma (STAD), testicular germ cell tumors (TGCT), thyroid carcinoma (THCA), thymoma (THYM), uterine corpus endometrial carcinoma (UCEC), uterine carcinosarcoma (UCS), and uveal melanoma (UVM), were downloaded from The Cancer Genome Atlas (TCGA; http://cancergenome.nih.gov) by UCSC Xena (https://xena.ucsc.edu/). To compare the CD93 expression between tumor and normal tissue, we additionally used the data of the Genotype Tissue Expression database (GTEx; https://commonfund.nih.gov/GTEx). The Tumor Immune Single-cell Hub database was used to examine the biodiversity of the tumor cell community and find heterogeneity in the TME.

### Survival Analysis and ROC Analysis

Four outcome parameters downloaded from TCGA, including overall survival (OS), disease-specific survival (DSS), disease-free interval (DFI), and progression-free interval (PFI), were incorporated in our study. To compare the overall survival (OS) rate for patients based on CD93 expression, we used Kaplan–Meier survival analysis using the log-rank test (*p* < 0.05). The survival and survminer R packages were used to draw survival curves. Univariate Cox analysis was used to evaluate the prognostic values across the 33 studied types of cancer (*p* < 0.05). The survival results were displayed with HR, 95% CI, and log-rank *p* values. We used the rms R package to construct baseline nomograms in KICH, LIHC, and UVM patients for clinical application and plotted receiver operating characteristic curves (ROC curves) in several types of cancer.

### Analysis of MMR, TMB, and MSI in Cancers

We surveyed the correlation between CD93 expression and several essential mismatch repair (MMR) genes, including the MutL homologous gene (MLH1) and the MutS homologous gene (MSH2, MSH6), and increased separation after meiosis (PMS2) and epithelial cell adhesion molecule (EPCAM) through TCGA. TMB is the total number of nonsynonymous mutations occurring in each coding region in the tumor genome (Chalmers et al., 2017). The higher the TMB is, the better clinical benefits obtained from immunotherapy (Meléndez et al., 2018; Marabelle et al., 2020). MSI, the condition of genetic hypermutability caused by the inactivation of mismatch repair genes, is another biomarker for immunotherapy (Ward et al., 2001; Boussios et al., 2019). The data were downloaded from UCSC Xena, and the correlation between CD93 expression and TMB and MSI was analyzed via Spearman’s rank correlation coefficient.

### TME and Immune Infiltration

The relationship of CD93 expression to TME was studied through evaluating the ratio of stromal and immune cells in tumors by estimate R packages, and the quantified results were displayed by tumor purity, stromal score, immune score, and estimate score. Specifically, stromal score and immune score stand for the stromal cells and the immunocyte infiltration level in tumor tissues, respectively. Estimate score was the sum of the preceding two, which infers the tumor purity. The estimated statistical significance and Spearman’s correlation coefficient were generated through correlation analysis. The Tumor Immune Estimation Resource (TIMER) database (https://cistrome.shinyapps.io/timer/) and the TIMER2.0 database (http://timer.cistrome.org/), which provided an integration of immune cells, including B cells, CD4^+^ T cells, CD8^+^ T cells, macrophages, neutrophils, and dendritic cells, for RNA sequencing samples from TCGA, were used to evaluate the abundance of tumor-infiltrating immune cells (TIICs) across diverse cancer types (Li et al., 2017). We next explored the relationship between CD93 and tumor purity as well as the abundance of TIICs by it. Finally, we explored the association between CD93 expression and immune-related genes, which includes HLA, immunostimulatory genes, immunosuppressive genes, chemokine, and chemokine receptor proteins, in 33 types of cancer.

### Enrichment Analysis

To explore the biological functions of CD93 across the pan-cancer, gene set enrichment analysis (GSEA) was performed between the low-CD93 and high-CD93 groups. Kyoto Encyclopedia of Genes and Genomes (KEGG) gene sets and HALLMARK gene sets were downloaded from the official GSEA website (https://www.gsea-msigdb.org/gsea/downloads.jsp). The enriched gene sets were selected based on *p* < 0.05, net enrichment score (NES) of greater than 1, and a false discovery rate (FDR) < 0.25.

### Statistical Analysis

All the data of gene expression were normalized by log_2_ transformation. The correlation analysis between the two variables used Spearman’s or Pearson’s test; *p* < 0.05 was considered significant. Student’s t-test and Kruskal–Wallis test were used for comparisons between two groups and for comparisons among >2 groups, respectively. All statistical analyses were processed by the R software (Version 4.0.2).

## Results

### Expression Analysis of CD93

**FIGURE 1:**
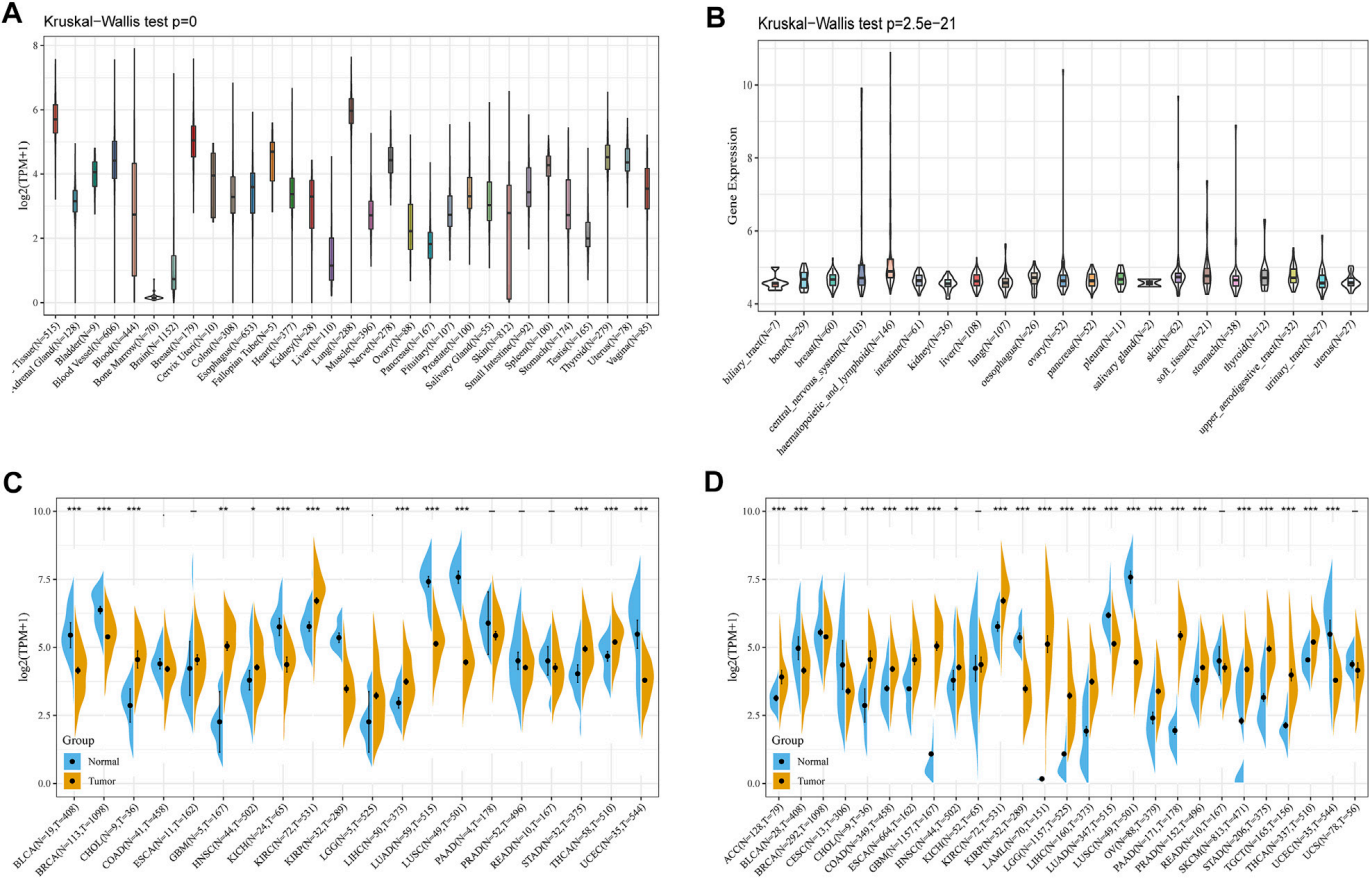
Expression analysis of CD93. **(A)** CD93 expression in various normal tissues. **(B)** CD93 expression in different tumor cell lines. **(C)** CD93 expression in the TCGA database. **(D)** Comparison of CD93 expressions between tumors and normal samples in the TCGA and the GTEx databases. *p < 0.05, **p < 0.01, ***p < 0.001.

To explore the basic expression of CD93, we first analyzed the mRNA expression of CD93 in 31 types of normal tissues through the Genotype-Tissue Expression (GTEx) portal (Kruskal–Wallis test, *p* < 0.001) (Figure 1A). The data showed that the expression of CD93 was the highest in lung and breast tissues, while it was the lowest in the bone marrow. However, it was low in most normal cell lines based on the data of the Cancer Cell Line Encyclopedia (CCLE) database (Kruskal–Wallis test, *p* < 0.001) (Figure 1B). Next, we evaluated its expression in TCGA pan-cancer and found it highly expressed in KIRC but low in LGG and LIHC (Figure 1C). Notably, there were obvious differences in CD93 expression between some tumor tissues and corresponding adjacent noncancerous tissues. Increased expression was found in CHOL, GBM, KIRC, LGG, LIHC, STAD, and THCA while decreased expression in BLCA, BRCA, KICH, KIRP, LUAD, LUSC, and UCEC. To fully analyze the differential expression of CD93 between normal and tumor samples as compelling a manner as possible, we integrated the data of the TCGA and GTEx databases and found that they were still differentially expressed in many cancers, most of which were consistent with the previous analysis (Figure 1D). More precisely, CD93 expression was significantly increased in ACC CHOL, COAD, ESCA, GBM, KIRC, LAML, LGG, LIHC, OV, PAAD, PRAD, SKCM, STAD, TGCT, and THCA, whereas it was downregulated in BLCA, KIRP, LUAD, LUSC, and UCEC. Now we have established that CD93 is expressed differently in different cancers.

### Prognostic and Diagnostic Value Analysis of CD93 in Pan-Cancer

**FIGURE 2:**
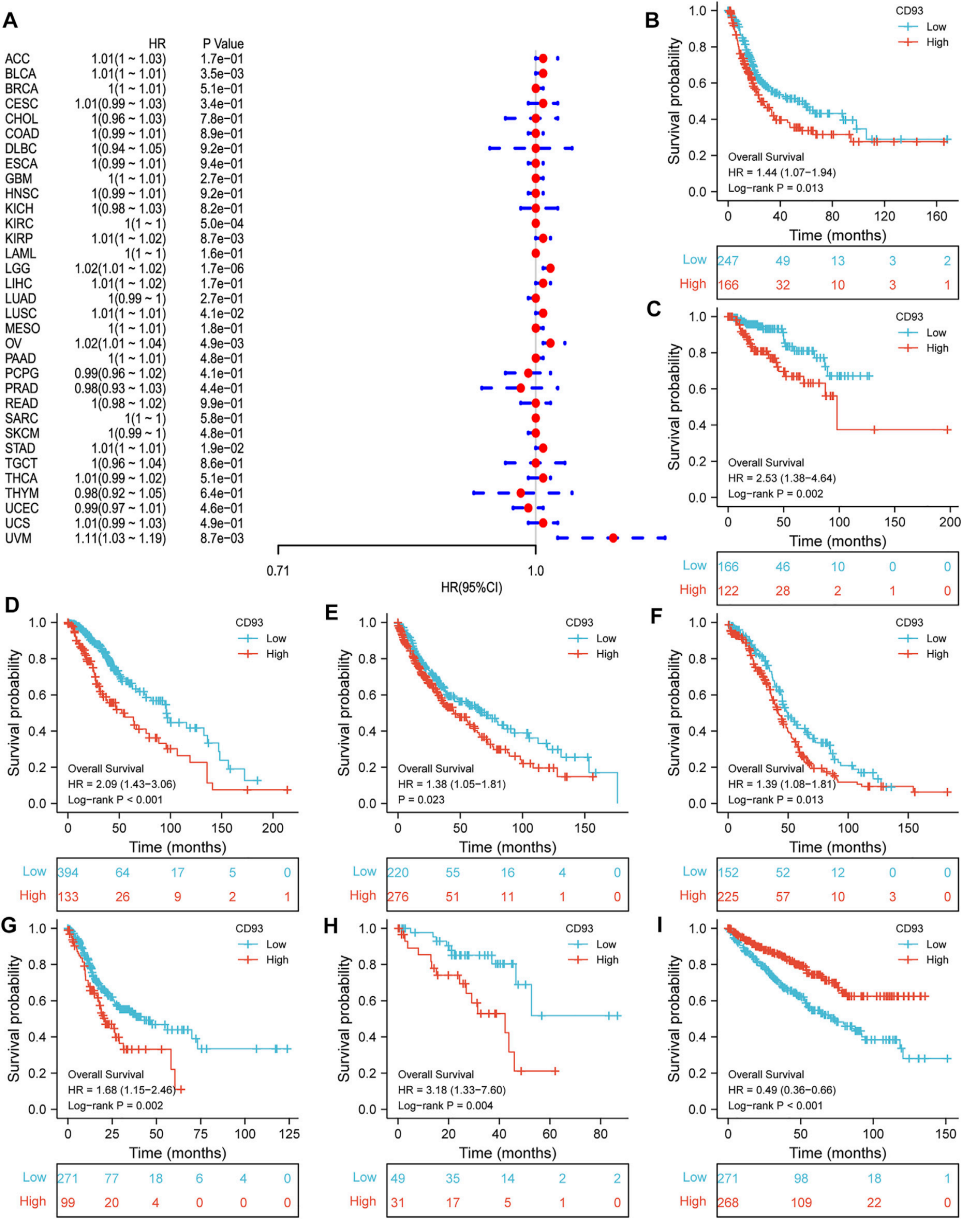
Association between CD93 and OS. **(A)** Forest plot of OS associations in 33 types of tumors. **(B–I)** Kaplan–Meier analysis of the association between CD93 and OS in BLCA, KIRP, LGG, LUSC, OV, STAD, UVM, and KIRC.

**FIGURE 3:**
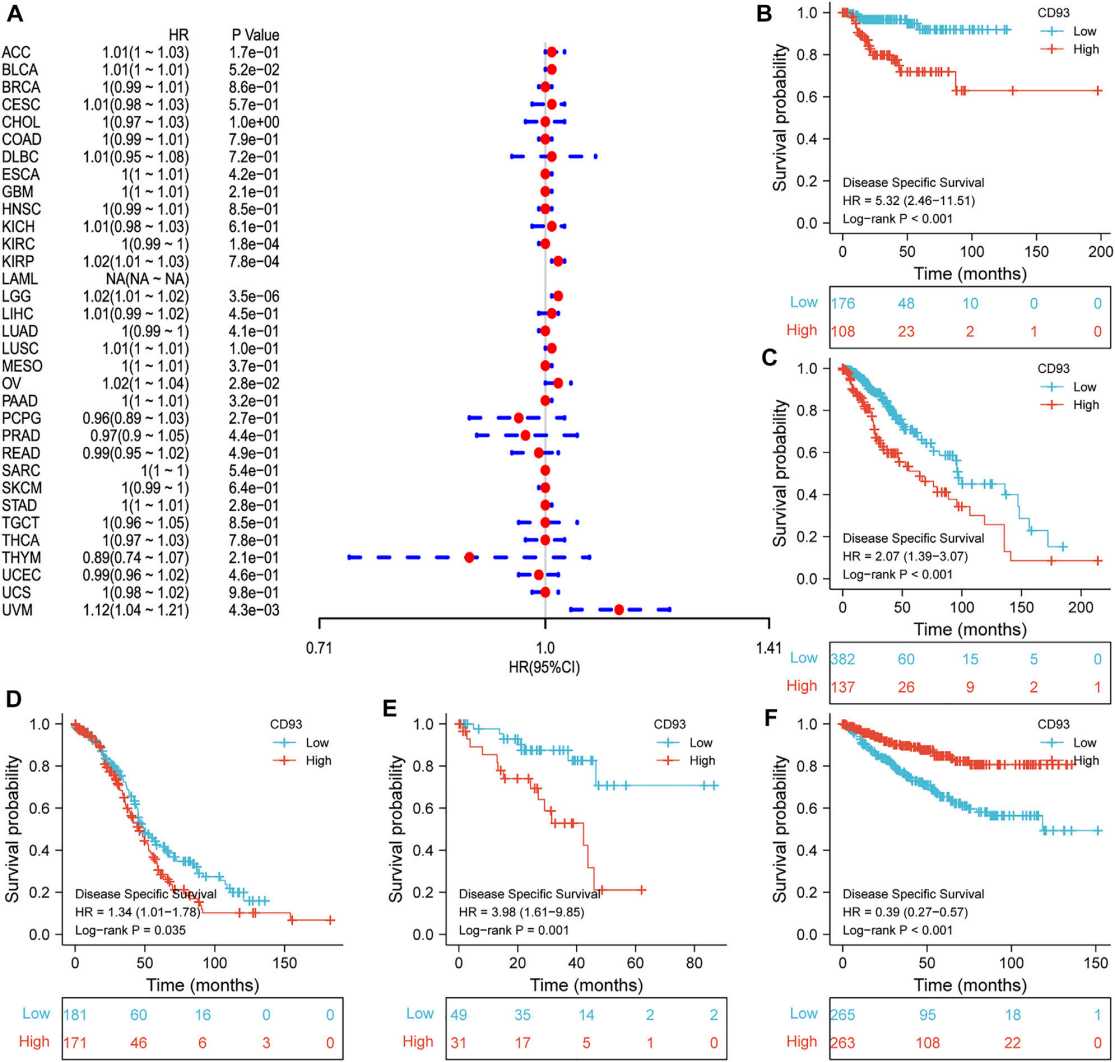
Association between CD93 and DSS. **(A)** Forest plot of DSS associations in 33 types of tumors. **(B–F)** Kaplan–Meier analysis of the association between the CD93 expression and DSS in KIRP, LGG, OV, UVM, and KIRC.

**FIGURE 4:**
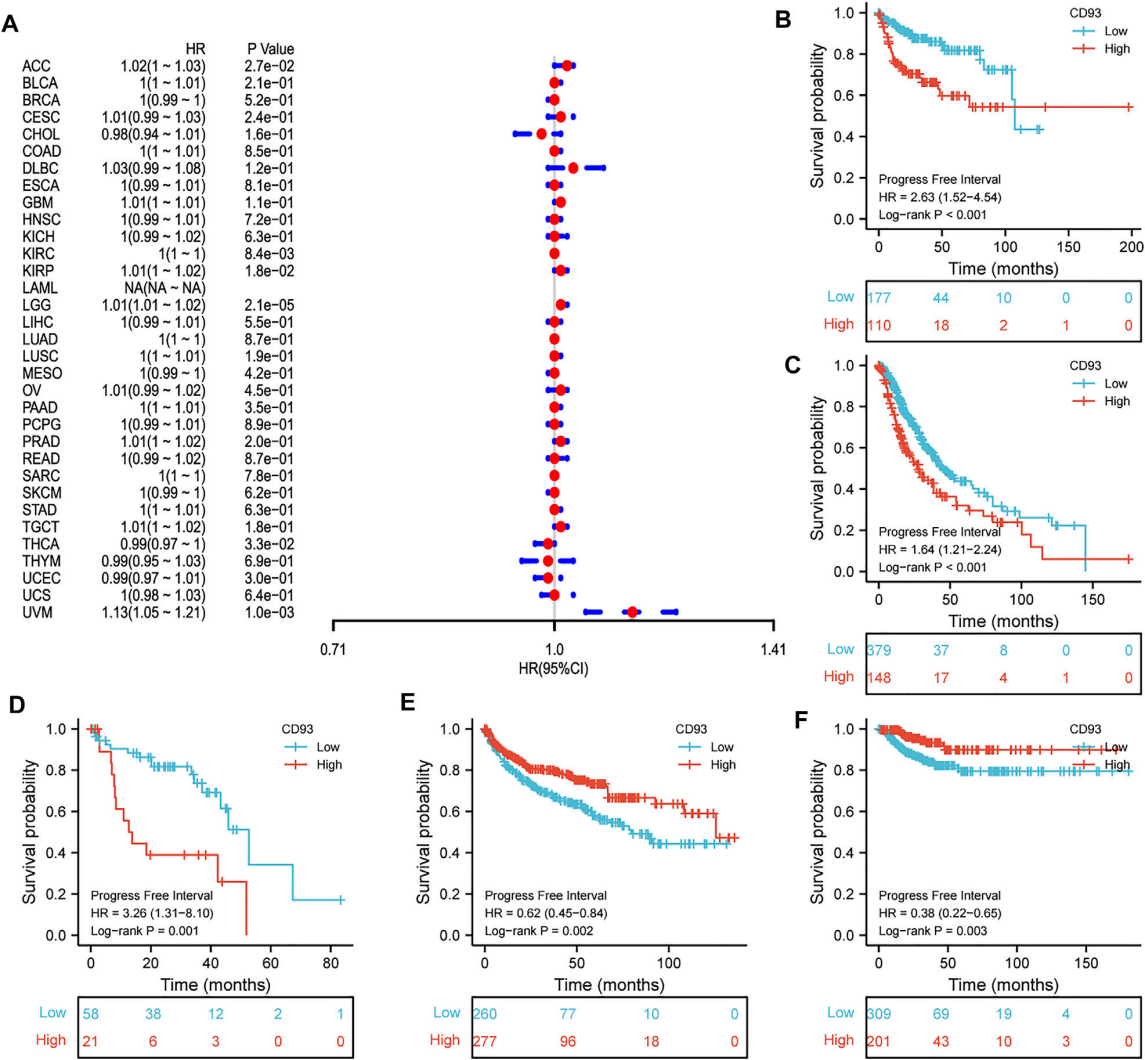
Association between CD93 expression and PFI. **(A)** Forest plot of PFI associations in 33 types of tumors. **(B–F)** Kaplan–Meier analysis of the association between the CD93 expression and PFI in KIRP, LGG, UVM, KIRC, and THCA.

Now that CD93 is known to be differentially expressed in certain cancers, we explored whether its expression is associated with the prognosis of different cancers. We then performed a survival association analysis for each cancer. The outcome parameters assessed were OS, DSS, PFI, and DFI. The analyses of OS by Cox regression indicated that CD93 acted as a general risk factor in BLCA (*p* = 0.0035), KIRP (*p* = 0.0087), LGG (*p* = 0.000017), LUSC (*p* = 0.041), OV (*p* = 0.0049), STAD (*p* = 0.019), and UVM (*p* = 0.0087) (Figure 2A). Interestingly, we obtained similar result and conclusion when we used the Kaplan–Meier survival analysis, revealing that CD93 was a high-risk factor in BLCA (*p* = 0.013), KIRP (*p* = 0.002), LGG (*p* < 0.001), LUSC (*p* = 0.023), OV (*p* = 0.013), STAD (*p* = 0.002), and UVM (*p* = 0.004) (Figures 2B–H). However, in this way, CD93 was a protective factor in KIRC (*p* < 0.001) (Figure 2I). Next, a correlation analysis was applied between CD93 expression and DSS to avoid the bias from people who did not die of cancer. Cox regression analysis showed that low CD93 expression levels were associated with poorer DSS in KIRP (*p* = 0.00078), LGG (*p* < 0.0001), OV (*p* = 0.028), and UVM (*p* = 0.0043), while they were associated with better DSS in KIRC (*p* < 0.0001) (Figure 3A). Similarly, the Kaplan–Meier analysis revealed worse prognostic impacts in KIRP (*p* < 0.001), LGG (*p* < 0.001), OV (*p* = 0.035), and UVM (*p* = 0.001) (Figures 3B–E), while better in KIRC (*p* < 0.001) (Figure 3F). The following results are about the expression of CD93 and PFI. Forest plots demonstrated that high levels of CD93 expression were closely related to adverse outcomes in patients with ACC (*p* = 0.027), KIRP (*p* = 0.018), LGG (*p* < 0.0001), and UVM (*p* < 0.0001), whereas that meant a contrary result in KIRC (*p* = 0.0084) and THCA (*p* = 0.033) (Figure 4A). In accordance with the Cox regression model, KM survival analysis showed that elevated CD93 expression was significantly related to a poorer PFI in KIRP (*p* < 0.001), LGG (*p* < 0.001), and UVM (*p* = 0.001) (Figures 4B–D), while it was significantly related to a better PFI in KIRC (*p* = 0.002) and THCA (*p* = 0.003) (Figures 4E,F). There was no obvious correlation between CD93 and DFI but UCEC (detailed information in Supplementary Figure S1).

To better predict the prognosis of KIRC, LIHC, and UVM patients in a clinic, a prognostic nomogram was developed by integrating gender, tumor stage, and CD93 expression into a univariate Cox regression model. A score based on the nomogram was calculated to predict the 1-, 3-, 5-, and 7-year OS for individual patients. The calibration plot showed that the nomogram performed well in predicting the OS of patients according to an ideal model. Additionally, AUC plotted for different durations of OS showed that CD93 expression was good at predicting the OS in KICH, LIHC, and UVM (Supplementary Figures S2–S4). We also demonstrated that CD93 can be a potential diagnostic biomarker in serval types of cancer via ROC curves (Supplementary Figure S5).

All of these data implied that the expression of CD93 was significantly associated with patient prognosis in multiple cancer types, especially in patients with KIRP, LGG, UVM, and KIRC.

### Correlations Between CD93 Expression and MMR, TMB, and MSI in Cancers

**FIGURE 5:**
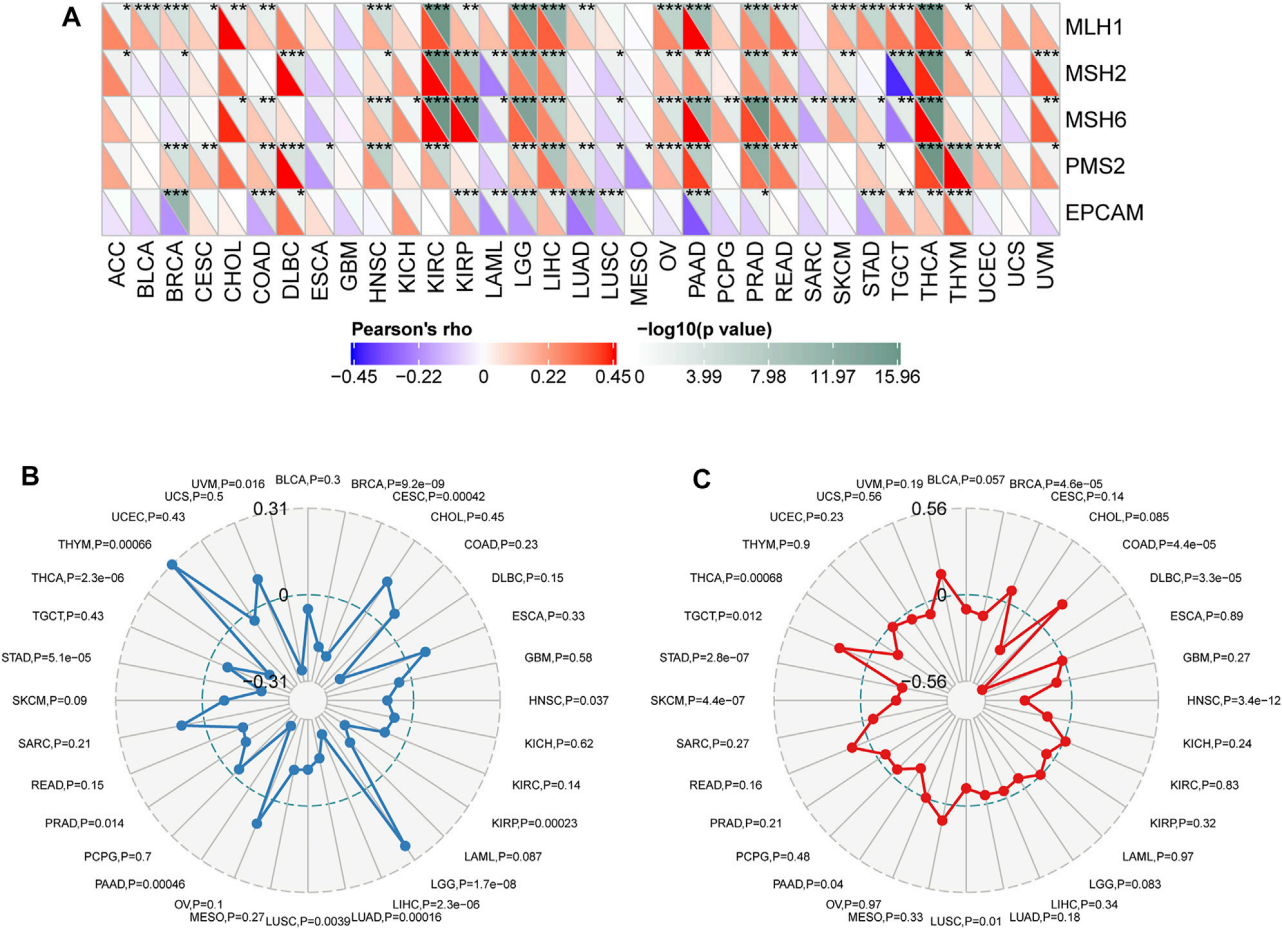
Correlations between CD93 expression and MMR, TMB, and MSI in cancers. **(A)** Correlations between CD93 expression and MMR genes (*p < 0.05, **p < 0.01, ***p < 0.001). **(B)** Correlations between CD93 expression and TMB across cancers. **(C)** Correlations between CD93 and MSI across cancers. The value in black reveals the range, and the curves in blue and red reveal the correlation coefficients.

Microsatellites (MSs), which are simple repetitive sequences of nucleotide bases, can generate errors during DNA replication, and MMR genes can recognize and fix this process. Tumors with defects in the MMR system are vulnerable to mutations in microsatellites, leading to high levels of MSI, leading in turn to the accumulation of mutation loads in cancer-related genes and the aggravation of TMB (Yarchoan et al., 2017; Joshi and Badgwell, 2021). We surveyed the correlation between CD93 expression and several essential MMR genes. CD93 expression was closely related to MMR genes in almost all 33 cancers but ACC, CESC, CHOL, ESCA, GBM, MESO, PCPG, SARC, and UCS (Figure 5A). Given that TMB status is gradually regarded as one of the promising pan-cancer biomarkers for forecasting ICB therapeutic effect, which has already been approved by the FDA (Chan et al., 2019), we checked the correlation between CD93 expression and TMB across cancers. As shown in Figure 5B, CD93 expression was positively related to TMB in LGG and THYM while negatively related to TMB in BRCA, CESC, HNSC, KIRP, LIHC, LUAD, LUSC, PAAD, PRAD, TAD, THCA, and UVMP. As another biomarker associated with ICI response, the association of MSI with CD93 expression was evaluated in our study (Figure 5C). Based on our analysis, the expression of CD93 had a positive relation with MSI in TGCT and COAD, while it indicated a negative relation in LUSC, PAAD, SKCM, STAD, THCA, BRCA, DLBC, and HNSC.

### CD93 and TME

**FIGURE 6:**
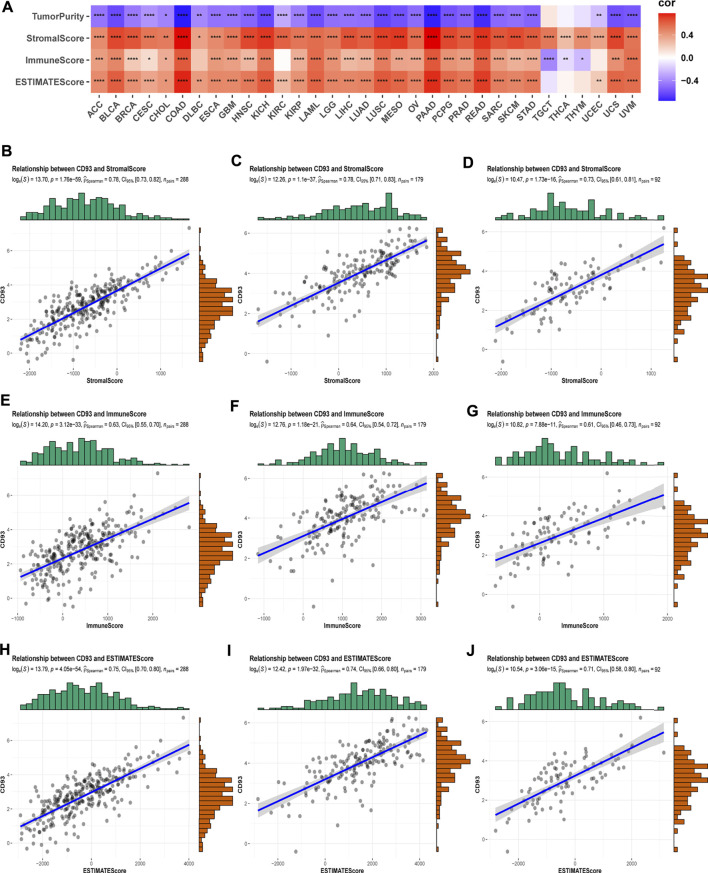
Correlation of CD93 expression with ImmuneScore and StromalScore in various cancers. **(A)** The Spearman’s correlation analysis of CD93 expression with ImmuneScore, StromalScores, and EstimateScores in 33 cancers (*p < 0.05, **p < 0.01, ***p < 0.001, ****p < 0.0001). Correlation of CD93 expression with three scores in COAD **(B–D)**, PAAD **(E–G)**, and READ **(H–J)**.

As we all know, TME plays a critical role in regulating malignant progression and modulating reactions to therapies, so strategies to therapeutically target TME have emerged as a promising approach for cancer treatment in recent years (Bejarano et al., 2021). To assess the association of CD93 expression and TME, we calculated the stromal, immune scores, ESTIMATE scores, and tumor purity in 33 types of cancer (Figure 6A). As shown in this study, the expression of CD93 is positively connected with stromal scores, immune scores, as well as ESTIMATE scores in the overwhelming majority of cancers but TGCT, THCA, THYM, and UCEC. However, our analysis indicated a negative contact between CD93 and tumor purity in most cancers. Specifically, CD93 was significantly associated with StromalScores and EstimateScores in COAD, BLCA, HNSC, KICH, LUSC, MESO, PAAD, READ, and USC and with immune scores in BLCA, COAD, KICH, LAML, LUSC, PAAD, READ, and UVM. At the same time, CD93 expression was closely related to tumor purity in ACC, BLCA, COAD, PAAD, and READ. We then visualized the top three cancers in detail among these analyses (Figures 6B–J). Additionally, we found varying degrees of heterogeneity between diverse landscapes of the TME by the Tumor Immune Single-cell Hub database. As shown in Supplementary Figure S6, we found that CD93 was extremely enriched in endothelial cells and mononuclear/macrophage subsets among all of these cancers. The expression of CD93 is concentrated malignantly in STAD, DC in STAD, and SKCM.

### The Relevance of CD93 Expression and Immune Infiltrates in Pan-Cancer

**FIGURE 7:**
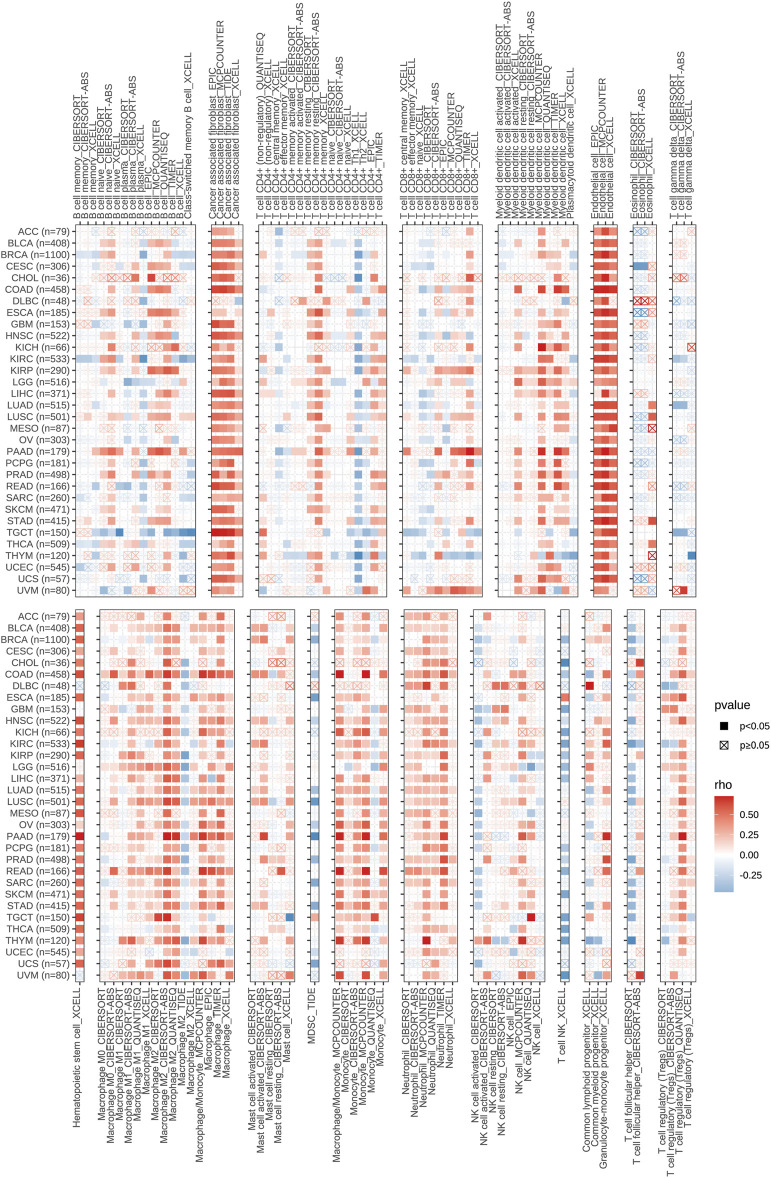
The relevance of CD93 expression and immune infiltrates in pan-cancer.

As one of the crucial parts of TME, immune cells make a great contribution to the homeostasis and evolution of TME (Vitale et al., 2019). To explore the correlation between CD93 expression and immune infiltration in human pan-cancer, we mainly focused on the association between CD93 and infiltrating immune cells by the TIMER2.0 database in various cancers (Figure 7). Notably, our results demonstrated that it had a strong positive contact between CD93 and CAFs, endothelial cells, myeloid dendritic cells, hematopoietic stem cells, mononuclear/macrophage subsets, and neutrophils while it had a negative correlation with Th1, MDSC, NK, and T follicular helper cells in almost all cancers we analyzed. Noticeably, CD93 was essentially enriched in CAFs, endothelial cells, and hematopoietic stem cells among these cancers. In addition, our data showed that increased CD93 expression was related to increased B cell infiltration in PAAD, KIRP, and ESCA, while it was related to decreased B cell infiltration in TGCT. CD93 expression was positively correlated with CD8^+^ T cells in UVM, PAAD, and KIRP while negatively in THYM. Concurrently, CD93 is enriched in neutrophils in THYM and DLBC, and mast cells in READ and PAAD. Compared with M0-M1-like macrophages, there was a stronger relationship between CD93 expression and M2-like macrophages, especially in UVM, UCS, TGCT, READ, PAAD, and COAD. We also analyzed the correlations between CD93 and B cells, CD8 + T cells, CD4 + T cells, macrophages, neutrophils, and dendritic cells in 32 types of cancer via the TIMER database. As shown in Supplementary Table S1, CD93 expression was mainly associated with six infiltrating immune cells in ACC, CHOL, COAD, HNSC, KIRP, LGG, LIHC, LUAD, LUSC, PAAD, PRAD, READ, SKCM, THCA, and UCEC. Among these cancers, CD93 was associated with six infiltrating immune cells of all cancers.

### Relationship Between CD93 and Immune Factors

**FIGURE 8:**
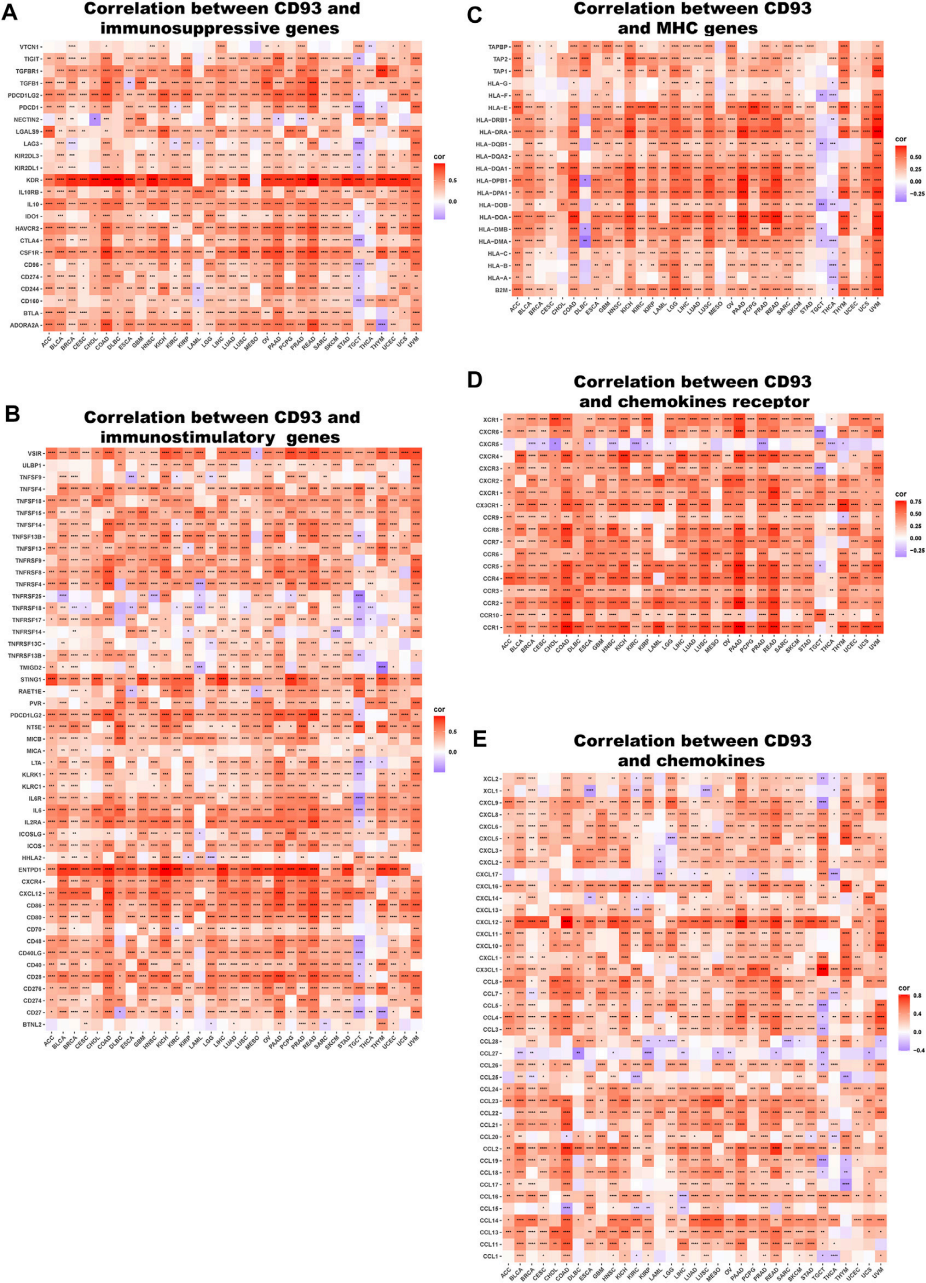
Relationship between CD93 and **(A)** immunosuppressive factors, **(B)** immunostimulatory factors, **(C)** MHC genes, **(D)** chemokine receptors, **(E)** and chemokines. *p < 0.05, **p < 0.01, ***p < 0.001, ****p < 0.0001.

We next explored the association between CD93 expression and immune-related genes, which includes HLA, immunostimulatory genes, immunosuppressive genes, chemokines, and chemokine receptor proteins, in 33 types of cancer. Our data showed that CD93 expression was positively correlated with almost all immune-related genes in nearly all cancers. As previously mentioned, elevated CD93 expression was related to increased immunosuppressive factors in all types of cancer except TGCT (Figure 8A). Noticeably, across the cancers we analyzed, the correlation was strongest between CD93 and KDR, and TGFBR1, TGFB1, PDCD1LG2, IL10, HAVCR2, CSF1R, and ADORA2A came second. Similar to immunosuppressive factors, CD93 expression was positively associated with immunostimulatory genes in all cancers we analyzed but TGCT (Figure 8B). In all analyzed cancers, ENTPD had the strongest correlation with CD93. Unlike other cancers, there was a more negative association between CD93 and immunostimulatory genes in TGCT. As shown by the heatmap presented in Figure 8C, there was a strong correlation between CD93 expression and almost all HLA genes in most cancers except for CHOL, DLBC, TGCT, and UCEC. Some of HLA genes, such as HLA-DMA, HLA-DOB, HLA-DQB1, and HLA-F, showed a negative correlation with CD93 in DLBC, TGCT, and THCA. In addition, we also analyzed the association between CD93 and chemokines as well as chemokine receptors (Figures 8D,E). The results showed that CD93 expression was positively correlated with chemokines and chemokine receptors, especially CXCL12 and CCR4.

### Functional Analysis of CD93

**FIGURE 9:**
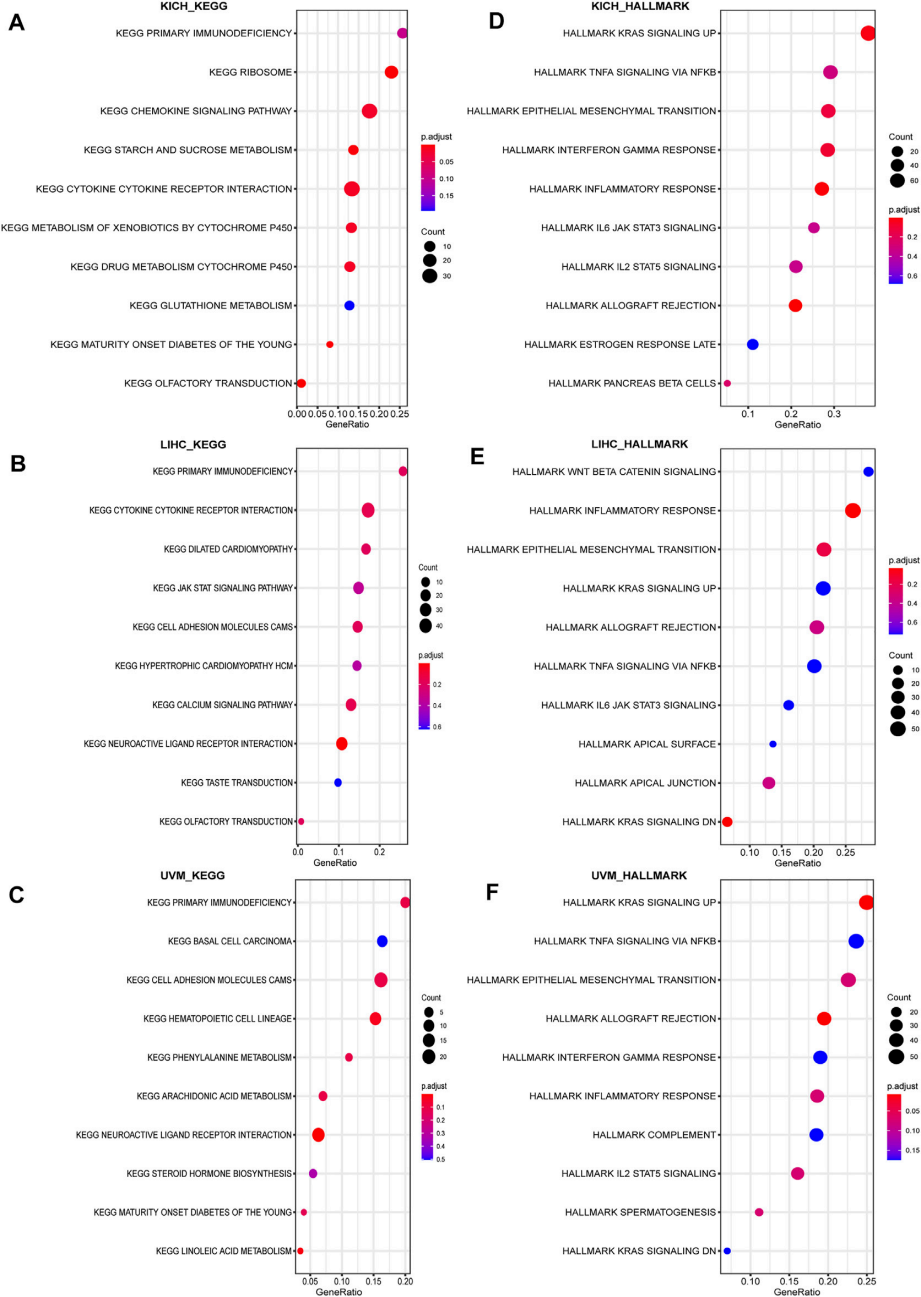
Result of GSEA. Different KEGG and HALLMARK pathways regulated in KICH **(A–B)**, LIHC **(B–D)**, and UVM **(E–F)**.

Lastly, GSEA was used to study the biological role of CD93 in different tumor tissues. Our data indicated that the top three positively enriched KEGG, from a pan-cancer perspective, in the elevated expression of CD93 were cytokine–cytokine/immunodeficiency. The HALLMARK enrichment term showed that CD93 expression was positively associated with the process of promoting cancers and inflammation, including interferon gamma response, inflammatory response, TNF signaling via NF-κB, complements, angiogenesis, EMT, IL-2-STAT5 signaling, kras signaling up, myc targets V2, and hedgehog signaling, in almost all cancers we analyzed. Here we showed the results in KICH (Figures 9A,B), LIHC (Figures 9C,D), and UVM (Figures 9E,F). To further validate the link between CD93 expression and other cancer-related biological functions in pan-cancer, we analyzed additional two biological roles of CD93 in various human tumors including autophagy, EMT, TGFB1, and Wnt pathway (Supplementary Figure S7). Interestingly, there was a positive correlation between CD93 and associated genes.

## Discussion

As a marker of tumor vasculature, the Group XIV family member endosialin was shown to regulate tumor growth and CD93 is homologous in structure to it (Nanda et al., 2006; Maia et al., 2011; Greenlee-Wacker et al., 2012). This suggests that CD93 may play key roles during cancer. However, despite the recent attention CD93 has received, its role in pan-cancer has remained unexplored prior to this investigation. In this study, we demonstrate that the differential expressions of CD93 in cancers help with prognosis estimation, the exploration and research of its mechanism, prediction of therapeutic effects, and the development of new treatments. It is conceivable to expect that the exploration of CD93 may therefore lead to major breakthroughs in the treatment of cancer, bringing great hope to those patients who have troubles with cancer diseases.

We first evaluated the expression levels and prognostic significance of CD93 in 33 types of cancers to explore its role across cancers and whether it can serve as a prognostic biomarker, which is a meaningful study to facilitate the translation of basic science to clinical studies. The results showed that elevated CD93 expression was found in CHOL, LGG, GBM, LIHC, and KIRC while low expression in KIRP, LUAD, and LUSC. Notably, the overexpression of CD93 was correlated with a worse prognosis in multiple cancers, such as LGG, OV, KIRP, and LUSC but KIRC. The expression of CD93 in tumors seems to make varying contributions among different types of cancer. Supporting that, CD93 overexpression was found in tumor vasculatures, and it influenced the survival of the host in PDAC, PNET, melanoma, and colon cancer (Langenkamp et al., 2015; Olsen et al., 2015; Sun et al., 2021). High CD93 levels were also found correlated with poor survival and increased proliferation capacity in leukemia (Pessoa Rodrigues and Akhtar, 2021). These findings clearly demonstrated the supporting role of CD93 in the prognosis of various cancers.

As a vital factor associated with the stability and integrity of genome, MMR has drawn considerable attention (Fishel, 2015; Russo et al., 2019). In addition, TMB and MSI are novel sensitive predictors of immunotherapy (Boland and Goel, 2010; Yarchoan et al., 2017; Lee et al., 2019). In our study, CD93 expression was enormously related to MMR genes in almost all cancers we analyzed. Additionally, CD93 also correlated with TMB and MSI in certain types of cancer. Then, the associations of CD93 expression with multiple checkpoint markers were compared across different cancer types. As expected, our data showed that CD93 was highly positively associated with almost all checkpoint genes in most cancers, which significantly indicated that CD93 may play an essential role in tumor immunity and may be the next promising marker in immunotherapy. Sun et al. observed that PD-L1 expression elevated in tumor tissues once anti-CD93 treatment and the growth of tumor were suppressed via the combination of CD93 and PD1 mAbs. Their results proved that blockade of the CD93 pathway can sensitize tumors to ICB therapy and facilitate cancer immunotherapy (Sun et al., 2021). This further demonstrated the importance of CD93 in immunotherapy.

Tumor cells exist in TME, which plays a central role in tumor initiation, development, metastasis, and clinical treatment and influences angiogenesis, tumor development, and immune escape (Lyssiotis and Kimmelman, 2017; Han et al., 2021; Hiam-Galvez et al., 2021). Our data showed that the expression of CD93 was positively connected with StromalScores, ImmuneScores, as well as ESTIMATE scores in human pan-cancer. Sun et al. (2021) also found that CD93 is selectively upregulated in tumor vasculature due to VEGF exposure in the TME, and its antibody can suppress tumor growth in mice, which hinted at the important role of CD93 in TME

However, despite its significant role in TME, the role of CD93 in immune infiltration needs to be further elucidated. Therefore, we further investigated on the role of CD93 in immune-cell infiltration levels in 33 types of cancer. Previous studies have reported that CD93 is expressed on a variety of cells such as neutrophils and endothelial cells (Greenlee-Wacker et al., 2011; Huang et al., 2021). In line with these findings, we showed that CD93 was enriched in CAFs, endothelial cells, and hematopoietic stem cells across different cancers. Supporting that, ECs are the primary cell type expressing CD93 revealed by published single-cell RNA sequencing datasets of multiple human tumor tissues (Sun et al., 2021). Single-cell transcriptomics revealed that CD93 can enrich HSC/MPP subsets with enhanced stem cell properties and regulate their development (Gao et al., 2021). In accordance with previous results on the role of CD93 in regulating immune infiltrates, we observed that CD93 was enriched in mononuclear/macrophage subsets, neutrophils, and B cells in some types of cancer. Consistently, CD93 was reported to be also expressed on several immune cell types, including monocytes, immature B cells, and platelets (Greenlee-Wacker et al., 2012). Based on the positive association of CD93 and M2 macrophages, which leads to an immunosuppressive phenotype, we can suppose that CD93 may exert an immune suppressive role in TME. More recently, numbers of works support the importance of CD93 expression in immune response during the development of cancers via alterations in immune cell phenotype, function, and infiltration (Galvagni et al., 2017; Lugano et al., 2018; Huang et al., 2021; Riether et al., 2021). For instance, CD93 was recently found to improve tumor vascular functions to promote T-cell infiltration and antitumor immunity after blockade of its pathway (Sun et al., 2021). In addition, we also found that CD93 expression was positively related to almost all immune-related genes, which are known to promote the growth of tumor cells and tumor angiogenesis as well as contribute to tumor metastasis (Li et al., 2021; Schaafsma et al., 2021) in nearly all cancers, which further demonstrated the importance of CD93 in the process of immune regulation.

To specifically address the function of CD93 in human pan-cancer, we performed enrichment analysis on CD93 and found a link between it and promoting cancers and inflammation. Concurrently, we have further validated the link between CD93 expression and autophagy, EMT, TGFB1, and the Wnt pathway. The finding that high expression of CD93 was mainly associated with inflammation and angiogenesis may imply that not only does CD93 exerts a crucial role in cancer, but it may be also important in inflammatory diseases and general homeostasis. Consistent with our data, CD93 was reported to be involved in apoptosis and inflammation and had a suggested role in angiogenesis, and thus involved in the development and dissemination of cancer (Olsen et al., 2015). CD93 also played roles in apoptosis, phagocytosis, inflammation, and cell adhesion (Nepomuceno et al., 1997; McGreal et al., 2002; Norsworthy et al., 2004). Interestingly, a key step during vascular maturation included the deposition of Multimerin-2, which acted not only as a substrate for pericyte adhesion but also as a central modifier of the expression pattern of important molecules regulating the crosstalk between ECs and pericytes and vascular stability (Fejza et al., 2021). However, as we all know, multimerin-2 is a known CD93 ligand (Galvagni et al., 2017; Khan et al., 2017). Consistently, IGFBP7, another known ligand for CD93, has been shown to promote EC angiogenesis via CD93 (Hooper et al., 2009; Komiya et al., 2014; Sun et al., 2021). These results suggest that CD93 is strongly associated with tumor invasion and metastasis.

These promising findings suggest that applying CD93 to the clinic is a feasible goal. Despite the provided new insights into CD93, more research will be required to understand how CD93 could modulate immune response. This would improve the efficiency of newer immunotherapy drugs, notably in combination with immunomodulatory therapies, which showed promising antitumor effects in some types of cancer in recent years. Nonetheless, our study identified a role for CD93 in regulating TME, influencing clinical prognosis and immunoregulation effect, particularly immune infiltration, and expanding our understanding of CD93 biology and tissue–immune system interaction, which may lead to new treatments for inflammatory diseases and cancer.

## Data Availability

The original contributions presented in the study are included in the article/Supplementary Material; further inquiries can be directed to the corresponding author.

## References

[B1] Bagaev A. Kotlov N. Nomie K. Svekolkin V. Gafurov A. Isaeva O. (2021). Conserved Pan-Cancer Microenvironment Subtypes Predict Response to Immunotherapy. Cancer Cell 39, 845–865. e7. 10.1016/j.ccell.2021.04.014 34019806

[B2] Barbera S. Lugano R. Pedalina A. Mongiat M. Santucci A. Tosi G. M. (2021). The C-type Lectin CD93 Controls Endothelial Cell Migration via Activation of the Rho Family of Small GTPases. Matrix Biol. 99, 1–17. 10.1016/j.matbio.2021.05.006 34062268

[B3] Bejarano L. Jordāo M. J. C. Joyce J. A. (2021). Therapeutic Targeting of the Tumor Microenvironment. Cancer Discov. 11, 933–959. 10.1158/2159-8290.CD-20-1808 33811125

[B4] Boland C. R. Goel A. (2010). Microsatellite Instability in Colorectal Cancer. Gastroenterology 138, 2073–2087. e3. 10.1053/j.gastro.2009.12.064 20420947 PMC3037515

[B5] Boussios S. Ozturk M. Moschetta M. Karathanasi A. Zakynthinakis-Kyriakou N. Katsanos K. (2019). The Developing story of Predictive Biomarkers in Colorectal Cancer. Jpm 9, 12. 10.3390/jpm9010012 PMC6463186 30736475

[B6] Bray F. Ferlay J. Soerjomataram I. Siegel R. L. Torre L. A. Jemal A. (2018). Global Cancer Statistics 2018: GLOBOCAN Estimates of Incidence and Mortality Worldwide for 36 Cancers in 185 Countries. CA: A Cancer J. Clinicians 68, 394–424. 10.3322/caac.21492 30207593

[B7] C. Greenlee-Wacker M. D. Galvan M. S. Bohlson S. (2012). CD93: Recent Advances and Implications in Disease. Cdt 13, 411–420. 10.2174/138945012799424651 22206251

[B8] Chalmers Z. R. Connelly C. F. Fabrizio D. Gay L. Ali S. M. Ennis R. (2017). Analysis of 100,000 Human Cancer Genomes Reveals the Landscape of Tumor Mutational burden. Genome Med. 9, 34. 10.1186/s13073-017-0424-2 28420421 PMC5395719

[B9] Chan T. A. Yarchoan M. Jaffee E. Swanton C. Quezada S. A. Stenzinger A. (2019). Development of Tumor Mutation burden as an Immunotherapy Biomarker: Utility for the Oncology Clinic. Ann. Oncol. 30, 44–56. 10.1093/annonc/mdy495 30395155 PMC6336005

[B10] Cokol M. Nair R. Rost B. (2000). Finding Nuclear Localization Signals. EMBO Rep. 1, 411–415. 10.1093/embo-reports/kvd092 11258480 PMC1083765

[B11] Fejza A. Poletto E. Carobolante G. Camicia L. Andreuzzi E. Capuano A. (2021). Multimerin-2 Orchestrates the Cross-Talk between Endothelial Cells and Pericytes: A Mechanism to Maintain Vascular Stability. Matrix Biol. Plus 11, 100068. 10.1016/j.mbplus.2021.100068 34435184 PMC8377000

[B12] Fishel R. (2015). Mismatch Repair. J. Biol. Chem. 290, 26395–26403. 10.1074/jbc.R115.660142 26354434 PMC4646297

[B13] Galvagni F. Nardi F. Spiga O. Trezza A. Tarticchio G. Pellicani R. (2017). Dissecting the CD93-Multimerin 2 Interaction Involved in Cell Adhesion and Migration of the Activated Endothelium. Matrix Biol. 64, 112–127. 10.1016/j.matbio.2017.08.003 28912033

[B14] Gao S. Shi Q. Zhang Y. Liang G. Kang Z. Huang B. (2021). Identification of HSC/MPP Expansion Units in Fetal Liver by Single-Cell Spatiotemporal Transcriptomics. Cell Res 32, 38–53. 10.1038/s41422-021-00540-7 34341490 PMC8724330

[B16] Greenlee-Wacker M. C. Briseño C. Galvan M. Moriel G. Velázquez P. Bohlson S. S. (2011). Membrane-associated CD93 Regulates Leukocyte Migration and C1q-Hemolytic Activity during Murine Peritonitis. J.I. 187, 3353–3361. 10.4049/jimmunol.1100803 PMC3169757 21849679

[B17] Han Z. Dong Y. Lu J. Yang F. Zheng Y. Yang H. (2021). Role of Hypoxia in Inhibiting Dendritic Cells by VEGF Signaling in Tumor Microenvironments: Mechanism and Application. Am. J. Cancer Res. 11, 3777–3793. 34522449 PMC8414384

[B18] Hiam-Galvez K. J. Allen B. M. Spitzer M. H. (2021). Systemic Immunity in Cancer. Nat. Rev. Cancer 21, 345–359. 10.1038/s41568-021-00347-z 33837297 PMC8034277

[B19] Hooper A. T. Shmelkov S. V. Gupta S. Milde T. Bambino K. Gillen K. (2009). Angiomodulin Is a Specific Marker of Vasculature and Regulates Vascular Endothelial Growth Factor-A-dependent Neoangiogenesis. Circ. Res. 105, 201–208. 10.1161/CIRCRESAHA.109.196790 19542015 PMC2936249

[B20] Huang J. Lee H.-y. Zhao X. Han J. Su Y. Sun Q. (2021). Interleukin-17D Regulates Group 3 Innate Lymphoid Cell Function through its Receptor CD93. Immunity 54, 673–686. e4. 10.1016/j.immuni.2021.03.018 33852831

[B21] Joshi S. S. Badgwell B. D. (2021). Current Treatment and Recent Progress in Gastric Cancer. CA A. Cancer J. Clin. 71, 264–279. 10.3322/caac.21657 PMC9927927 33592120

[B22] Khan K. A. Naylor A. J. Khan A. Noy P. J. Mambretti M. Lodhia P. (2017). Multimerin-2 Is a Ligand for Group 14 Family C-type Lectins CLEC14A, CD93 and CD248 Spanning the Endothelial Pericyte Interface. Oncogene 36, 6097–6108. 10.1038/onc.2017.214 28671670 PMC5671938

[B23] Komiya E. Sato H. Watanabe N. Ise M. Higashi S. Miyagi Y. (2014). Angiomodulin, a Marker of Cancer Vasculature, Is Upregulated by Vascular Endothelial Growth Factor and Increases Vascular Permeability as a Ligand of Integrin α V β 3. Cancer Med. 3, 537–549. 10.1002/cam4.216 24737780 PMC4101744

[B24] Langenkamp E. Zhang L. Lugano R. Huang H. Elhassan T. E. A. Georganaki M. (2015). Elevated Expression of the C-type Lectin CD93 in the Glioblastoma Vasculature Regulates Cytoskeletal Rearrangements that Enhance Vessel Function and Reduce Host Survival. Cancer Res. 75, 4504–4516. 10.1158/0008-5472.CAN-14-3636 26363010

[B25] Lee D.-W. Han S.-W. Bae J. M. Jang H. Han H. Kim H. (2019). Tumor Mutation burden and Prognosis in Patients with Colorectal Cancer Treated with Adjuvant Fluoropyrimidine and Oxaliplatin. Clin. Cancer Res. 25, 6141–6147. 10.1158/1078-0432.CCR-19-1105 31285374

[B26] Li L. Yao W. Yan S. Dong X. Lv Z. Jing Q. (2021). Pan-cancer Analysis of Prognostic and Immune Infiltrates for CXCs. Cancers 13, 4153. 10.3390/cancers13164153 34439306 PMC8392715

[B27] Li T. Fan J. Wang B. Traugh N. Chen Q. Liu J. S. (2017). TIMER: A Web Server for Comprehensive Analysis of Tumor-Infiltrating Immune Cells. Cancer Res. 77, e108–e110. 10.1158/0008-5472.CAN-17-0307 29092952 PMC6042652

[B28] Lugano R. Vemuri K. Yu D. Bergqvist M. Smits A. Essand M. (2018). CD93 Promotes β1 Integrin Activation and Fibronectin Fibrillogenesis during Tumor Angiogenesis. J. Clin. Invest. 128, 3280–3297. 10.1172/JCI97459 29763414 PMC6063507

[B29] Lyssiotis C. A. Kimmelman A. C. (2017). Metabolic Interactions in the Tumor Microenvironment. Trends Cel Biol. 27, 863–875. 10.1016/j.tcb.2017.06.003 PMC5814137 28734735

[B30] Maia M. DeVriese A. Janssens T. Moons M. Lories R. J. Tavernier J. (2011). CD248 Facilitates Tumor Growth via its Cytoplasmic Domain. BMC Cancer 11, 162. 10.1186/1471-2407-11-162 21549007 PMC3107809

[B31] Marabelle A. Fakih M. Lopez J. Shah M. Shapira-Frommer R. Nakagawa K. (2020). Association of Tumour Mutational burden with Outcomes in Patients with Advanced Solid Tumours Treated with Pembrolizumab: Prospective Biomarker Analysis of the Multicohort, Open-Label, Phase 2 KEYNOTE-158 Study. Lancet Oncol. 21, 1353–1365. 10.1016/S1470-2045(20)30445-9 32919526

[B32] Masiero M. Simões F. C. Han H. D. Snell C. Peterkin T. Bridges E. (2013). A Core Human Primary Tumor Angiogenesis Signature Identifies the Endothelial Orphan Receptor ELTD1 as a Key Regulator of Angiogenesis. Cancer Cell 24, 229–241. 10.1016/j.ccr.2013.06.004 23871637 PMC3743050

[B33] McGreal E. P. Ikewaki N. Akatsu H. Morgan B. P. Gasque P. Human C. (2002). Human C1qRp Is Identical with CD93 and the mNI-11 Antigen but Does Not Bind C1q. J. Immunol. 168, 5222–5232. 10.4049/jimmunol.168.10.5222 11994479

[B34] Meléndez B. Van Campenhout C. Rorive S. Remmelink M. Salmon I. D’Haene N. (2018). Methods of Measurement for Tumor Mutational burden in Tumor Tissue. Transl. Lung Cancer Reslung Cancer Res. 7, 661–667. 10.21037/tlcr.2018.08.02 PMC6249625 30505710

[B35] Nanda A. Karim B. Peng Z. Liu G. Qiu W. Gan C. (2006). Tumor Endothelial Marker 1 (Tem1) Functions in the Growth and Progression of Abdominal Tumors. Proc. Natl. Acad. Sci. 103, 3351–3356. 10.1073/pnas.0511306103 16492758 PMC1413931

[B36] Nepomuceno R. R. Henschen-Edman A. H. Burgess W. H. Tenner A. J. (1997). cDNA Cloning and Primary Structure Analysis of C1qRP, the Human C1q/MBL/SPA Receptor that Mediates Enhanced Phagocytosis *In Vitro* . Immunity 6, 119–129. 10.1016/s1074-7613(00)80419-7 9047234

[B38] Norsworthy P. J. Fossati-Jimack L. Cortes-Hernandez J. Taylor P. R. Bygrave A. E. Thompson R. D. (2004). Murine CD93 (C1qRp) Contributes to the Removal of Apoptotic Cells *In Vivo* but Is Not Required for C1q-Mediated Enhancement of Phagocytosis. J. Immunol. 172, 3406–3414. 10.4049/jimmunol.172.6.3406 15004139

[B39] Olsen R. S. Lindh M. Vorkapic E. Andersson R. E. Zar N. Löfgren S. (2015). CD93 Gene Polymorphism Is Associated with Disseminated Colorectal Cancer. Int. J. Colorectal Dis. 30, 883–890. 10.1007/s00384-015-2247-1 26008729 PMC4471320

[B40] Passaro A. Stenzinger A. Peters S. (2020). Tumor Mutational burden as a Pan-Cancer Biomarker for Immunotherapy: The Limits and Potential for Convergence. Cancer Cell 38, 624–625. 10.1016/j.ccell.2020.10.019 33171127

[B41] Pessoa Rodrigues C. Akhtar A. (2021). Differential H4K16ac Levels Ensure a Balance between Quiescence and Activation in Hematopoietic Stem Cells. Sci. Adv. 7. 10.1126/sciadv.abi5987 PMC8346211 34362741

[B42] Riether C. Radpour R. Kallen N. M. Bürgin D. T. Bachmann C. Schürch C. M. (2021). Metoclopramide Treatment Blocks CD93-Signaling-Mediated Self-Renewal of Chronic Myeloid Leukemia Stem Cells. Cel Rep. 34, 108663. 10.1016/j.celrep.2020.108663 33503440

[B43] Russo M. Crisafulli G. Sogari A. Reilly N. M. Arena S. Lamba S. (2019). Adaptive Mutability of Colorectal Cancers in Response to Targeted Therapies. Science 366, 1473–1480. 10.1126/science.aav4474 31699882

[B44] Samstein R. M. Lee C.-H. Shoushtari A. N. Hellmann M. D. Shen R. Janjigian Y. Y. (2019). Tumor Mutational Load Predicts Survival after Immunotherapy across Multiple Cancer Types. Nat. Genet. 51, 202–206. 10.1038/s41588-018-0312-8 30643254 PMC6365097

[B45] Schaafsma E. Fugle C. M. Wang X. Cheng C. (2021). Pan-cancer Association of HLA Gene Expression with Cancer Prognosis and Immunotherapy Efficacy. Br. J. Cancer 125, 422–432. 10.1038/s41416-021-01400-2 33981015 PMC8329209

[B46] Sun Y. Chen W. Torphy R. J. Yao S. Zhu G. Lin R. (2021). Blockade of the CD93 Pathway Normalizes Tumor Vasculature to Facilitate Drug Delivery and Immunotherapy. Sci. Transl. Med. 13. 10.1126/scitranslmed.abc8922 PMC8749958 34321321

[B47] Vitale I. Manic G. Coussens L. M. Kroemer G. Galluzzi L. (2019). Macrophages and Metabolism in the Tumor Microenvironment. Cel Metab. 30, 36–50. 10.1016/j.cmet.2019.06.001 31269428

[B48] Ward R. Meagher A. Tomlinson I. O'Connor T. Norrie M. Wu R. (2001). Microsatellite Instability and the Clinicopathological Features of Sporadic Colorectal Cancer. Gut 48, 821–829. 10.1136/gut.48.6.821 11358903 PMC1728324

[B49] Yarchoan M. Hopkins A. Jaffee E. M. (2017). Tumor Mutational burden and Response Rate to PD-1 Inhibition. N. Engl. J. Med. 377, 2500–2501. 10.1056/NEJMc1713444 29262275 PMC6549688

