# Supplementary material for: CD93 Correlates With Immune Infiltration and Impacts Patient Immunotherapy Efficacy: A Pan-Cancer Analysis
Source: Front Cell Dev Biol. 2022 Feb 15;10:817965. doi: 10.3389/fcell.2022.817965 (PMC8886047; doi:10.3389/fcell.2022.817965)
Supplement: Supplementary file 1 [file DataSheet1.docx]

Supplementary Material


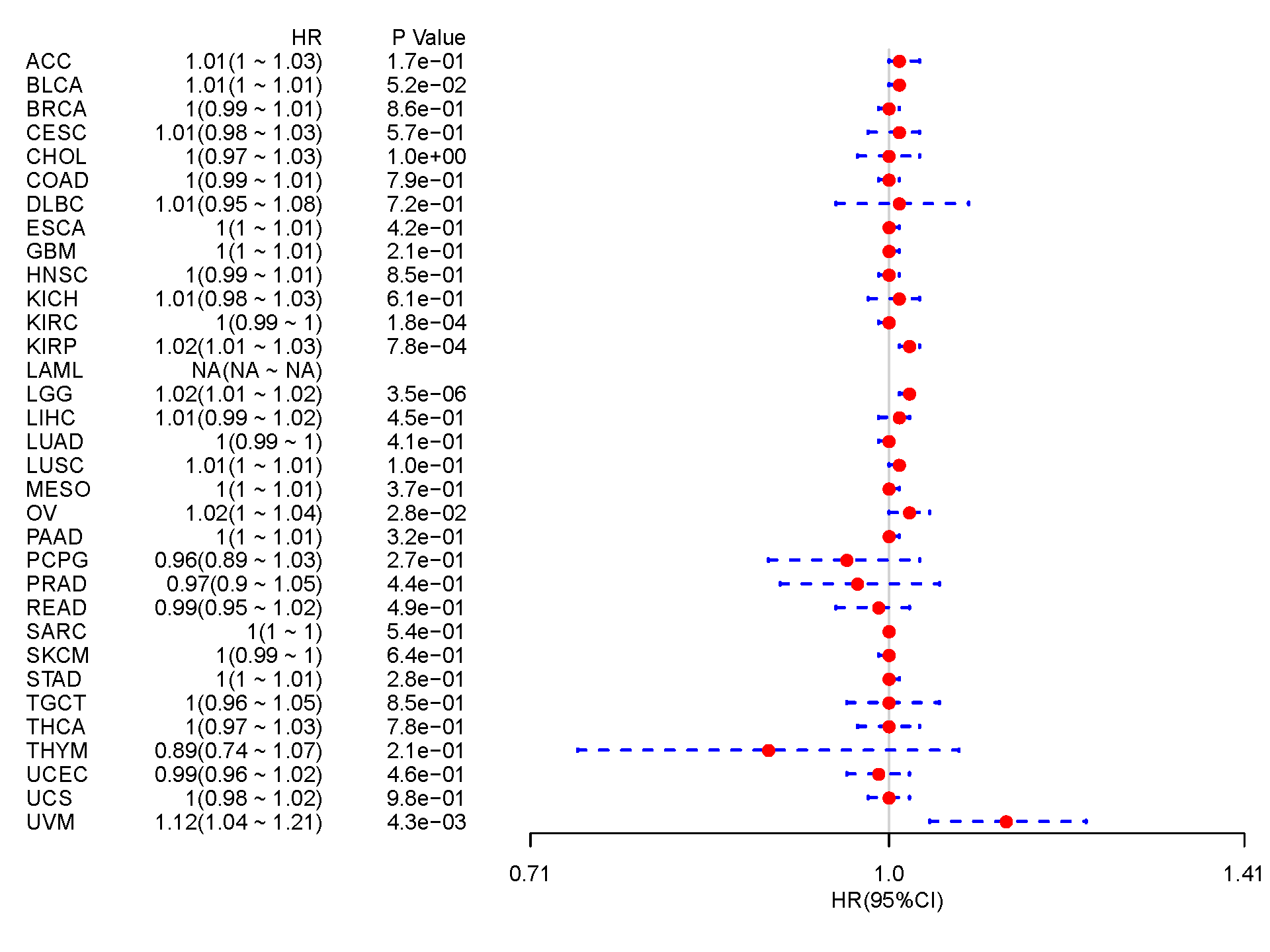


**Supplementary Figure 1.** Forest plot denoting the association between CD93 and disease-free interval (DFI).


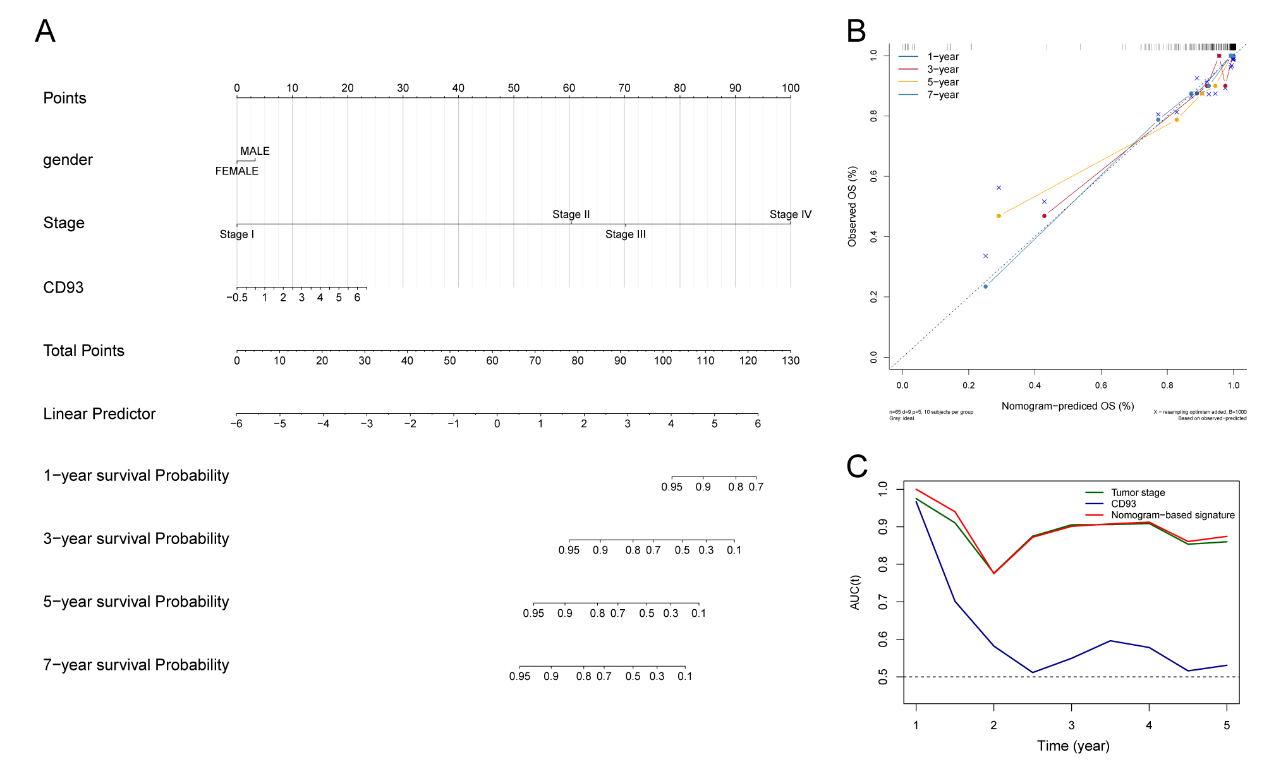


**Supplementary Figure 2.** Construction of a CD93-based prognostic prediction model in KICH. (A) Nomogram for the prediction of survival rates at 1-, 3-, 5-, 7-year in KICH. (B) Calibration curves of the nomogram. (C) Time-dependent ROC curve for validation.


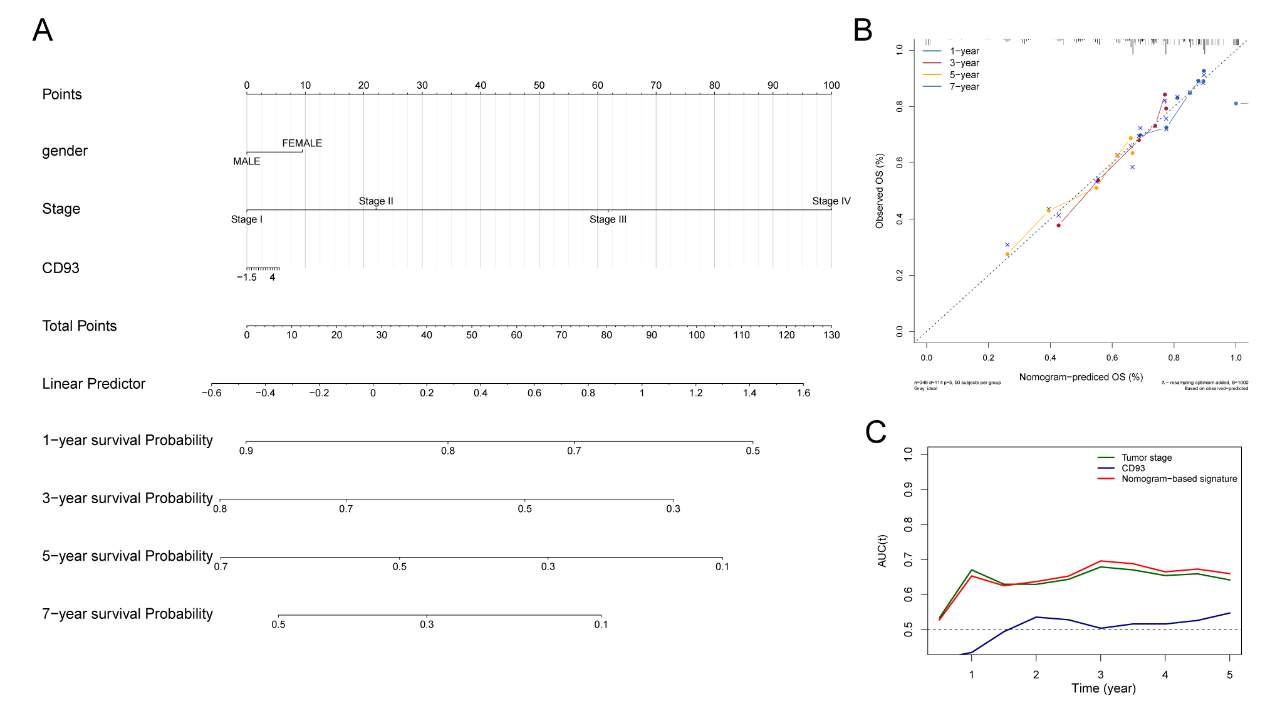


**Supplementary Figure 3.** Construction of a CD93-based prognostic prediction model in LIHC. (A) Nomogram for the prediction of survival rates at 1-, 3-, 5-, 7-year in LIHC. (B) Calibration curves of the nomogram. (C) Time-dependent ROC curve for validation.


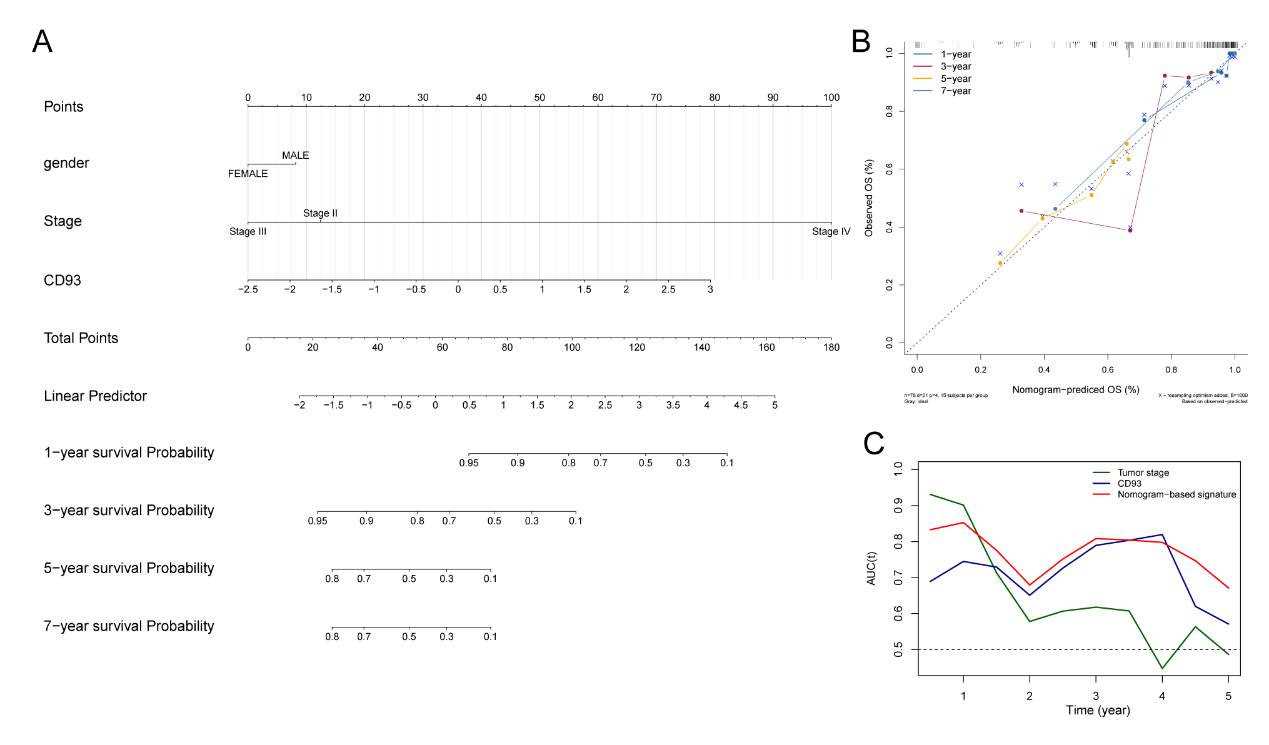
 **Supplementary Figure 4.** Construction of a CD93-based prognostic prediction model in UVM. (A) Nomogram for the prediction of survival rates at 1-, 3-, 5-, 7-year in UVM. (B) Calibration curves of the nomogram. (C) Time-dependent ROC curve for validation.


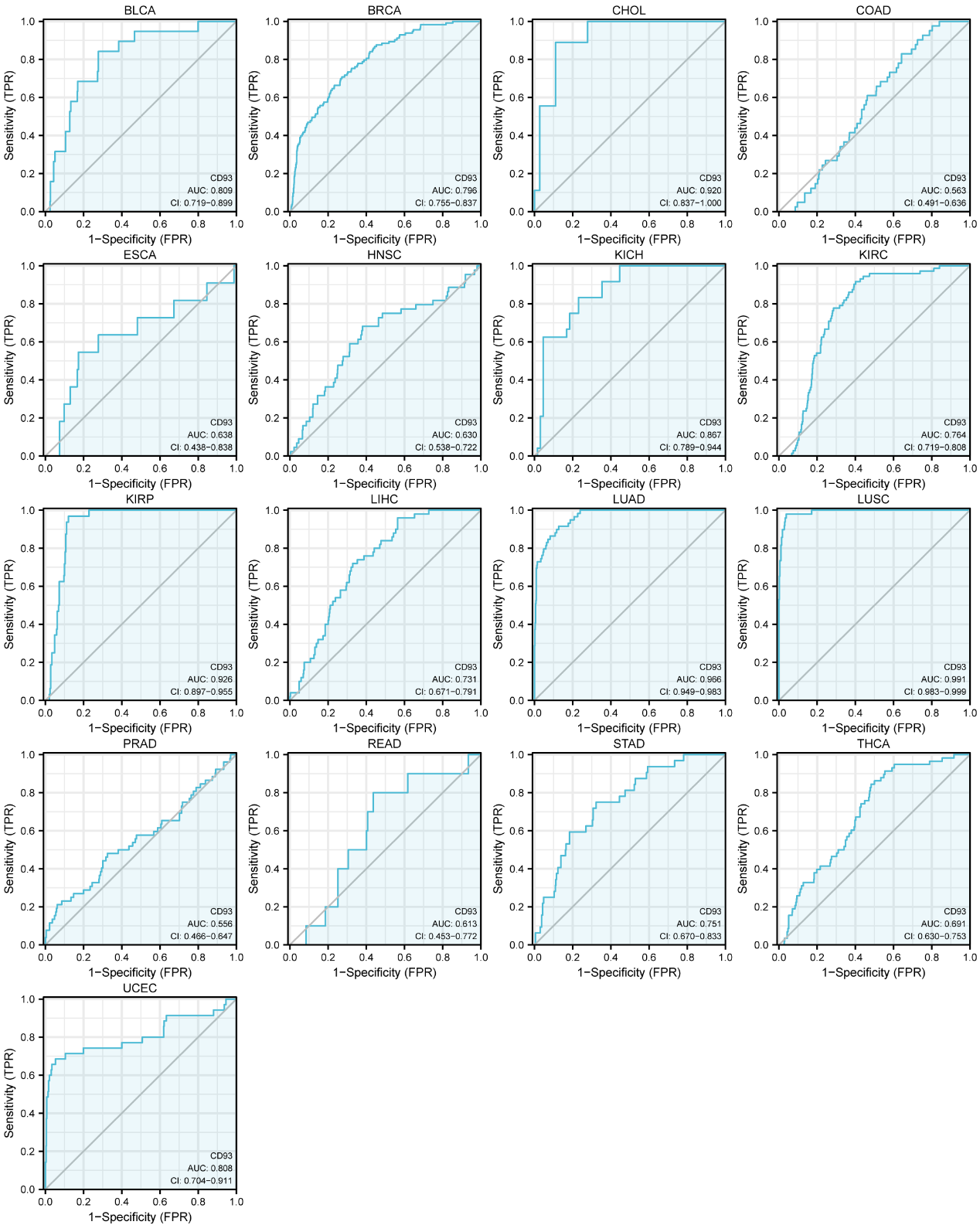


**Supplementary Figure 5.** ROC curves of CD93 in serval types of cancer in TCGA.





**Supplementary Figure 6.** CD93 expression heterogeneity between the diverse landscapes of TME as per the Tumor Immune Single-cell Hub database


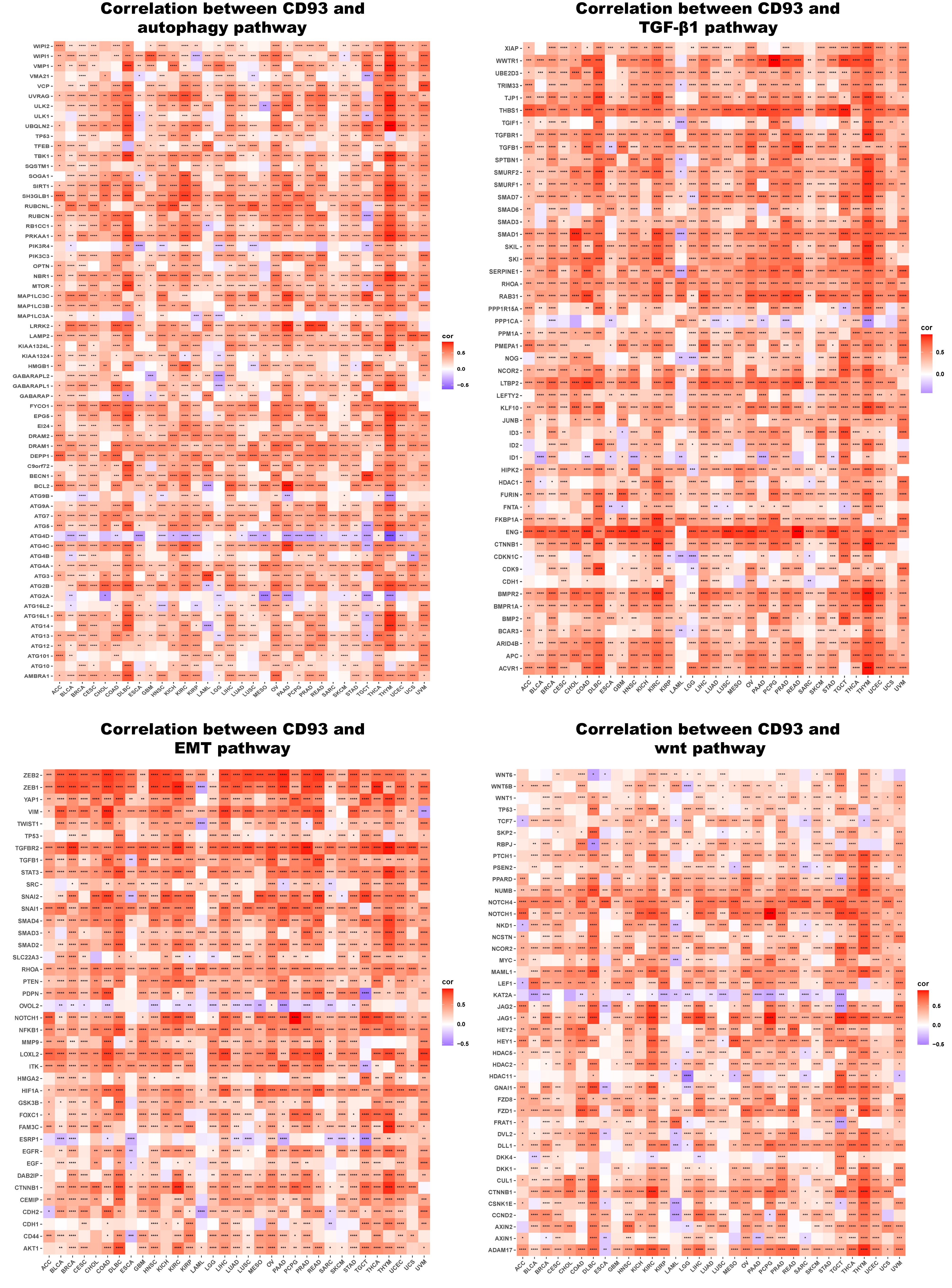


**Supplementary Figure 7.** Correlation between CD93 and autophagy, TGF-β1, EMT, wnt pathways.

**Supplementary Table 1.** Correlation between CD93 and six immune cells in timer database.

| **Cancer** | **B Cell** | **CD8+ T Cell** | **CD4+ T Cell** | **Macrophage** | **Neutrophil** | **Dendritic Cell** |
| --- | --- | --- | --- | --- | --- | --- |
| ACC | 0.434 | 0.405 | 0.424 | 0.411 | 0.544 | 0.685 |
| BLCA | 0.052 | 0.224 | 0.063 | 0.425 | 0.265 | 0.158 |
| BRCA | 0.055 | 0.459 | 0.328 | 0.474 | 0.326 | 0.312 |
| CESC | 0.176 | 0.086 | 0.226 | 0.328 | 0.184 | 0.246 |
| CHOL | 0.627 | 0.455 | 0.464 | 0.547 | 0.662 | 0.449 |
| COAD | 0.158 | 0.367 | 0.565 | 0.657 | 0.647 | 0.631 |
| DLBC | 0.094 | -0.054 | 0.203 | 0.155 | 0.150 | -0.085 |
| ESCA | 0.286 | 0.128 | 0.282 | 0.436 | 0.154 | -0.018 |
| GBM | 0.061 | -0.162 | 0.127 | 0.173 | 0.122 | 0.397 |
| HNSC | 0.241 | 0.248 | 0.505 | 0.509 | 0.344 | 0.487 |
| KICH | 0.465 | 0.392 | -0.025 | 0.417 | 0.124 | 0.563 |
| KIRC | 0.057 | 0.376 | 0.456 | 0.445 | 0.439 | 0.318 |
| KIRP | 0.451 | 0.502 | 0.224 | 0.185 | 0.383 | 0.551 |
| LGG | 0.270 | 0.390 | 0.350 | 0.428 | 0.460 | 0.464 |
| LIHC | 0.253 | 0.302 | 0.467 | 0.501 | 0.526 | 0.480 |
| LUAD | 0.119 | 0.230 | 0.278 | 0.471 | 0.434 | 0.384 |
| LUSC | 0.234 | 0.335 | 0.494 | 0.611 | 0.514 | 0.584 |
| MESO | 0.290 | 0.199 | 0.124 | 0.520 | 0.160 | 0.332 |
| OV | 0.013 | 0.131 | 0.151 | 0.316 | 0.302 | 0.270 |
| PAAD | 0.392 | 0.612 | 0.323 | 0.770 | 0.631 | 0.677 |
| PCPG | -0.060 | 0.364 | 0.433 | 0.330 | 0.581 | 0.475 |
| PRAD | 0.450 | 0.581 | 0.335 | 0.506 | 0.626 | 0.648 |
| READ | 0.178 | 0.282 | 0.299 | 0.404 | 0.515 | 0.459 |
| SARC | -0.020 | 0.220 | 0.125 | 0.386 | 0.387 | 0.120 |
| SKCM | 0.178 | 0.227 | 0.249 | 0.517 | 0.382 | 0.321 |
| STAD | -0.009 | 0.204 | 0.421 | 0.510 | 0.325 | 0.379 |
| TGCT | -0.309 | -0.206 | 0.023 | 0.225 | 0.039 | -0.108 |
| THCA | 0.407 | -0.296 | 0.378 | 0.370 | 0.232 | 0.184 |
| THYM | -0.043 | -0.154 | -0.283 | -0.021 | 0.512 | -0.222 |
| UCEC | 0.138 | 0.297 | 0.232 | 0.223 | 0.370 | 0.352 |
| UCS | 0.069 | 0.089 | 0.210 | 0.596 | 0.338 | 0.519 |
| UVM | 0.044 | 0.401 | -0.216 | -0.050 | -0.166 | 0.055 |
